# Safety and efficacy of viltolarsen in ambulatory and nonambulatory males with Duchenne muscular dystrophy

**DOI:** 10.1038/s41598-024-70783-y

**Published:** 2024-10-08

**Authors:** Amy D. Harper, Haluk Topaloglu, Eugenio Mercuri, Vasiliy Suslov, Liwen Wu, Cigdem Y. Ayanoglu, Michael Tansey, Michelle L. Previtera, Robert A. Crozier, Leslie Magnus, Paula R. Clemens

**Affiliations:** 1 Children’s Hospital of Richmond at Virginia Commonwealth University, Richmond, VA USA; 2 Department of Pediatrics, Yeditepe University, Istanbul, Turkey; 3 Gemelli Hospital Catholic University Foundation, Rome, Italy; 4 Saint Petersburg State Paediatric Medical University, St Petersburg, Russia; 5 Hunan Children’s Hospital, Hunan, China; 6 NS Pharma, Inc., Paramus, NJ USA; 7 Department of Neurology, University of Pittsburgh School of Medicine, 3550 Terrace St, Pittsburgh, PA 15261 USA; 8 Department of Veterans Affairs Medical Center, Pittsburgh, PA USA

**Keywords:** Neuromuscular disease, Pediatric research, Phase II trials, Neuromuscular disease

## Abstract

Duchenne muscular dystrophy (DMD) is an X-linked recessive disease characterized by mutations in the dystrophin gene, causing motor and pulmonary function decline. Viltolarsen is indicated for patients with dystrophin gene mutations amenable to exon 53 skipping. Here, we report safety, motor function, and the first pulmonary function results from the open-label, phase II Galactic53 trial of viltolarsen (NCT04956289). Male participants aged ≥ 8 years with DMD received 80 mg/kg intravenous viltolarsen once weekly for 48 weeks. Results from participants receiving viltolarsen were compared with an external control cohort group-matched for multiple variables. All treatment-emergent adverse events were mild or moderate, 4 were considered treatment-related, and no participants discontinued. Participants receiving viltolarsen experienced clinically meaningful benefits in pulmonary function with higher percent predicted forced vital capacity and higher peak cough flow at Week 49 compared with the control cohort for both ambulatory and nonambulatory participants. Viltolarsen also stabilized upper limb motor function over the Treatment Period. These results support viltolarsen as an important part of the treatment armamentarium for both ambulatory as well as nonambulatory patients with DMD.

## Introduction

Duchenne muscular dystrophy (DMD) is an X-linked recessive disorder that affects approximately 1 in every 3600 to 9300 live male births^1^. DMD is caused by mutations in the dystrophin gene, the majority of which result in reading frame shifts leading to loss of functional dystrophin protein production^2–4^. Boys with DMD are typically diagnosed by 5 years of age due to impaired motor function and delayed attainment of age-appropriate motor milestones^5^. Patients with DMD have severely affected restrictive pulmonary function as the disease progresses, with the eventual need for assisted ventilation^6^.

Both pulmonary and motor function continue to decline with age. Loss of ambulation occurs around early adolescence with the treatment of glucocorticoids. Most patients die due to cardiac and/or pulmonary events in the third or fourth decade of life^7–10^.

Glucocorticoids are the standard of care to slow disease progression but do not restore dystrophin protein levels^11,12^. This leaves an unmet need for safe and effective therapies that increase dystrophin expression levels and provide additional functional benefit in DMD. Dystrophin deficiency typically caused by in-frame gene mutations, known as Becker muscular dystrophy, results in a shortened, internally deleted dystrophin protein expressed in muscle^13,14^. Therefore, Becker muscular dystrophy can be less severe than DMD, with slower progression and longer survival^13–15^. Exon skipping therapies for patients with DMD with an out-of-frame dystrophin gene deletion offer the potential for restoration of the open reading frame of the dystrophin pre-mRNA. An antisense oligonucleotide binds to the target exon in pre-mRNA, which induces skipping of that exon during splicing to restore the open reading frame. This promotes the production of shortened, internally deleted dystrophin protein with retention of essential functional portions, similar to the naturally occurring dystrophin in Becker muscular dystrophy^9^. It is estimated that 55% to 80% of DMD mutations are amenable to exon skipping, and approximately 8% to 10% of all mutations are amenable to exon 53 skipping^3^.

Viltolarsen, a phosphorodiamidate morpholino oligomer (PMO) therapy that is commercially available in the United States and Japan, is an antisense oligonucleotide indicated for the treatment of DMD in patients who have a confirmed mutation of the dystrophin gene that is amenable to exon 53 skipping^16,17^. In preclinical studies, viltolarsen treatment promoted a dose-dependent increase in exon 53 skipping during pre-mRNA splicing and elevated dystrophin protein levels in cells^18^.

In previous clinical studies, viltolarsen significantly increased dystrophin protein levels, stabilized motor function over 4 years, and was well tolerated^19–21^. In both a phase II and long-term extension study, most treatment-emergent adverse events (TEAEs) with viltolarsen were mild or moderate, and only 1 was considered treatment-related^19,20^. However, these studies did not evaluate the effects of viltolarsen on pulmonary function. In addition, safety and motor function have not been evaluated in nonambulatory patients with DMD in a viltolarsen clinical trial. Here we report safety and changes in pulmonary and motor function from the phase II Galactic53 trial (NCT04956289; registered 09/07/2021) of viltolarsen in ambulatory and nonambulatory males, ages ≥ 8 years, with DMD amenable to exon 53 skipping.

## Results

### Participants

From July 2021 to July 2023, 21 males with DMD were screened, and 20 participants were enrolled to receive once-weekly 80 mg/kg viltolarsen for 48 weeks. Baseline characteristics were well-matched for ambulatory and nonambulatory participants between the viltolarsen group and the control group from the Cooperative International Neuromuscular Research Group (CINRG) Duchenne Natural History Study (DNHS) (Table 1).

**Table 1:** Baseline demographics and clinical characteristics of ambulatory and nonambulatory participants.

Participant characteristic	Ambulatory		Nonambulatory	
	Viltolarsen 80 mg/kg/week n = 10	CINRG DNHS control cohort n = 25	Viltolarsen 80 mg/kg/week n = 10	CINRG DNHS control cohort n = 23
Age, years, mean (SD)	9.8 (1.6)	10.0 (2.3)	15.8 (6.4)	15.6 (3.5)
Range﻿	8–12	8–17	8–26	10–21
Race, n (%)				
White	6 (60)	22 (88)	9 (90)	16 (70)
Asian	4 (40)	2 (8)	1 (10)	5 (22)
Black or African American	0	1 (4)	0	1 (4)
Other	0	0	0	1 (4)
Ethnicity, n (%)				
Not hispanic or Latino	10 (100)	21 (84)	10 (100)	22 (96)
Weight, kg, mean (SD)	33.1 (10.4)	33.4 (8.8)	49.4 (19.5)	54.3 (21.4)
Height^a^, cm, mean (SD)	127.2 (6.6)	126.6 (10.5)	148.1 (14.5)	150.3 (11.4)
Body mass index, kg/m^2^, mean (SD)	20.1 (4.2)	20.6 (3.4)	21.9 (6.2)	23.5 (7.5)
Baseline steroid, n (%)				
Deflazacort	5 (50)	14 (56)	5 (50)	18 (78)
Prednisone or prednisolone	5 (50)	11 (44)	4 (40)	5 (22)
None	0	0	1 (10)	0
Assessments, mean (SD)				
FVC%p	83.5 (9.3)	85.3 (18.2)	58.8 (14.6)	59.7 (17.2)
PCF, L/min	170.4 (59.2)	145.0 (50.6)	151.2 (99.2)	186.6 (57.6)
PUL 2.0 total score	38.1 (2.2)	NA^b^	23.5 (9.3)	NA^b^
PUL 2.0 midlevel elbow score	16.3 (0.7)	NA^b^	9.9 (5.1)	NA^b^

*CINRG* Cooperative International Neuromuscular Research Group, *DNHS* Duchenne Natural History Study, *FVC%p* percent predicted forced vital capacity, *NA* not available, *PCF* peak cough flow, *PUL* performance of upper limb, *SD* standard deviation.

^a^If the standing height could not be obtained, it was derived based on ulnar length.

^b^Data not collected.

The mean (standard deviation [SD]) ages of ambulatory participants receiving viltolarsen and the CINRG DNHS controls were 9.8 years (1.6) and 10.0 years (2.3), respectively. For ambulatory participants treated with viltolarsen and the control cohort, the mean (SD) weights were 33.1 kg (10.4) and 33.4 kg (8.8), respectively, and the mean heights (SD) were 127.2 cm (6.6) and 126.6 cm (10.5), respectively. All ambulatory participants receiving viltolarsen (100%) and the CINRG DNHS control cohort (100%) were on a stable dose of glucocorticoids.

Baseline pulmonary function between ambulatory participants receiving viltolarsen and the CINRG DNHS control cohort was comparable with a mean (SD) percent predicted forced vital capacity (FVC%p) of 83.5% (9.3) and 85.3% (18.2), respectively; the mean (SD) peak cough flow (PCF) was 170.4 L/min (59.2) and 145.0 L/min (50.6) for participants receiving viltolarsen and the CINRG DNHS control cohort, respectively. For ambulatory participants receiving viltolarsen, the baseline mean (SD) Performance of Upper Limb (PUL) 2.0 total score was 38.1 (2.2). The PUL test was not part of the CINRG DNHS protocol.

Consistent with the natural progression of DMD, nonambulatory boys were generally more symptomatic than ambulatory boys as well as being older, heavier, and taller. The mean (SD) ages of nonambulatory participants receiving viltolarsen and the CINRG DNHS controls were 15.8 years (6.4) and 15.6 years (3.5), respectively. For nonambulatory participants treated with viltolarsen and the control cohort, the mean (SD) weights were 49.4 kg (19.5) and 54.3 kg (21.4), respectively, and the mean (SD) heights were 148.1 cm (14.5) and 150.3 cm (11.4), respectively. All but one nonambulatory participant receiving viltolarsen (90%) and all participants in the CINRG DNHS control cohort (100%) were on a stable dose of glucocorticoids.

Baseline pulmonary function between nonambulatory participants receiving viltolarsen and the CINRG DNHS control cohort was comparable, with a mean (SD) FVC%p of 58.8% (14.6) and 59.7% (17.2), respectively; the mean (SD) PCF was 151.2 L/min (99.2) and 186.6 L/min (57.6) for participants receiving viltolarsen and the CINRG DNHS control cohort, respectively. One 26-year-old participant treated with viltolarsen reported use of nighttime ventilation prior to study entry. No other participants reported use of nighttime ventilation, and cough assist devices were not used during the study. For nonambulatory participants receiving viltolarsen, the baseline mean (SD) PUL 2.0 total score was 23.5 (9.3).

### Safety

In the safety population, 95% (19/20) of participants receiving viltolarsen reported TEAEs (Table 2); all TEAEs were mild or moderate in severity, and the most common (≥ 15% of participants) were COVID-19 infection (n = 6), headache (n = 4), hematuria (n = 4), nasopharyngitis (n = 3), and upper respiratory tract infection (n = 3) (Table 3). Four participants experienced treatment-related TEAEs, and none were considered severe (hematuria [n = 2], allergic reaction [n = 1], and hypertension [n = 1]). There were no serious adverse events, deaths, or discontinuations due to TEAEs. No induction of serum anti-dystrophin or anti-viltolarsen antibodies was detected in participants receiving viltolarsen. No clinically meaningful trends were identified for any chemistry, hematology, coagulation, urinalysis, conventional urine chemistry, or urine cytology parameters over the duration of the study.

**Table 2:** Summary of TEAEs in participants receiving viltolarsen.

Participants with the following	Viltolarsen 80 mg/kg/week N = 20^a^
Any TEAE, n (%)	19 (95)
Any treatment-related TEAE^b^, n (%)	4 (20)
Discontinuation due to TEAE, n (%)	0
Any serious drug-related TEAE, n (%)	0
Death, n (%)	0

*TEAE* treatment-emergent adverse event.

^a^Includes both ambulatory and nonambulatory participants.

^b^Hematuria (n = 2), allergic reaction (n = 1), and hypertension (n = 1). Both cases of hematuria consisted of reports of red blood cells seen on urinalysis, and no further analysis was conducted; both participants continued viltolarsen infusions, and the hematuria resolved without treatment. The allergic reaction occurred during Week 46 of treatment and presented as runny nose, sneezing, itching, and rash. The participant was treated with oral cetirizine, and symptoms resolved. This participant continued to receive a dose of oral cetirizine prior to further viltolarsen infusions, and there were no recurrences of the symptoms. The participant with hypertension had 1 reading of elevated blood pressure during the first infusion without subsequent occurrences.

**Table 3:** TEAEs in > 1 participant receiving viltolarsen.

Preferred term	Viltolarsen 80 mg/kg/week N = 20﻿
COVID-19 infection	6 (30)
Headache	4 (20)
Hematuria	4 (20)
Nasopharyngitis	3 (15)
Upper respiratory tract infection	3 (15)
Diarrhea	2 (10)
Food poisoning	2 (10)
Influenza	2 (10)
Joint injury	2 (10)
Pain in extremity	2 (10)
Pyrexia	2 (10)
Rhinitis	2 (10)

Data shown as n (%). Reported terms were coded using the Medical Dictionary for Regulatory Activities version 26.0.

*COVID-19* coronavirus disease 2019, *TEAE* treatment-emergent adverse event.

### Efficacy

**Fig. 1:**
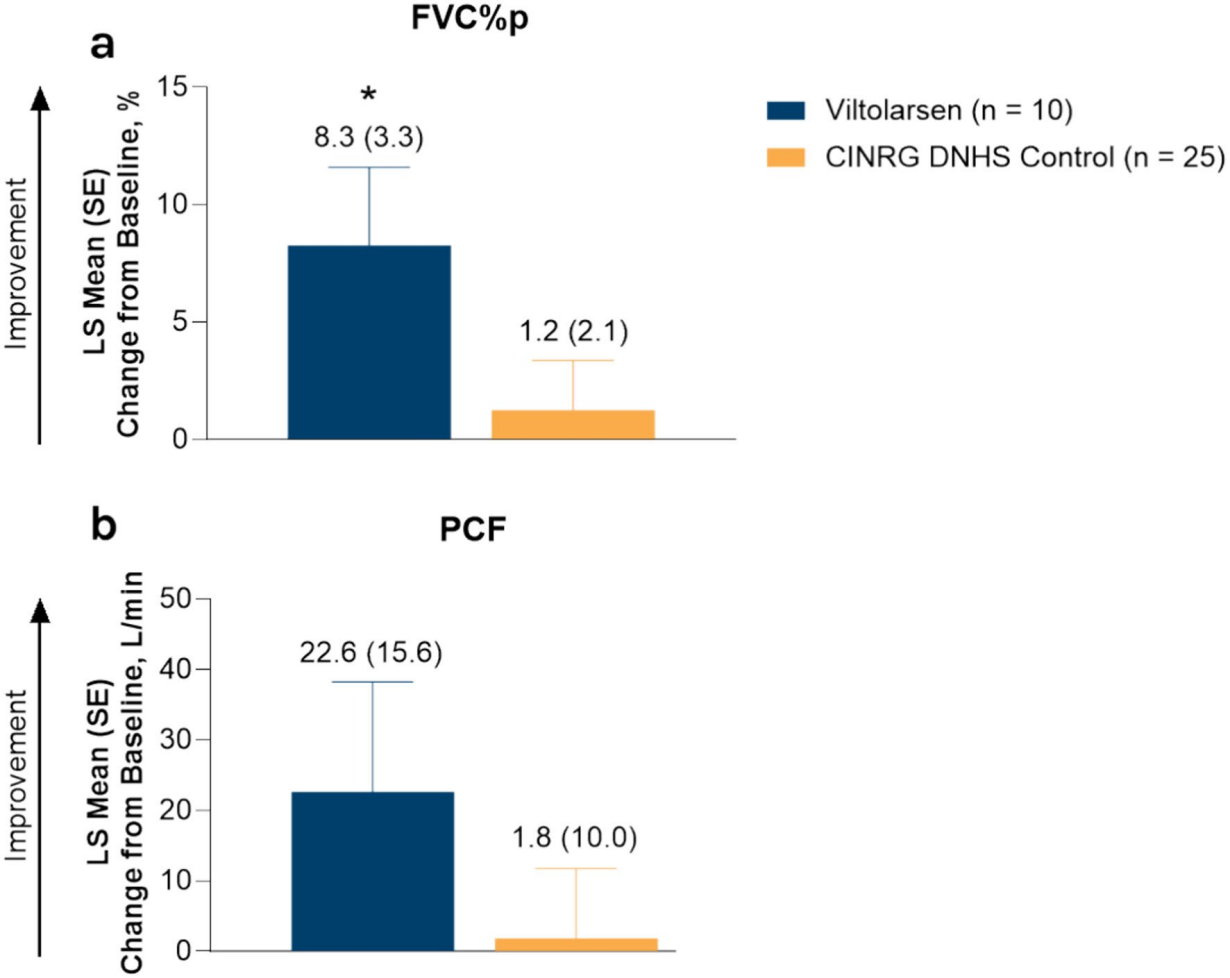
LS mean change from baseline in FVC%p (**a**) and PCF (**b**) in ambulatory participants at Week 49. The values above the bars are LS mean (SE). All *P*-values are nominal. (**a**) *P = 0.02 vs baseline. *CINRG* Cooperative International Neuromuscular Research Group, *DNHS* Duchenne Natural History Study, *FVC%p* percent predicted forced vital capacity, *LS* least squares, *PCF* peak cough flow, *SE* standard error.

For ambulatory participants treated with viltolarsen, the least squares (LS) mean change from baseline (standard error [SE]) in FVC%p was significant at Week 49 (8.3% [3.3], *P* = 0.02) (Fig. 1a). In addition, 90% (9/10) of ambulatory participants receiving viltolarsen had an increase or stabilization in FVC%p from baseline, and all participants maintained FVC%p values > 50% at Week 49. For ambulatory participants, the LS mean change from baseline (SE) in PCF was numerically higher for those receiving viltolarsen vs CINRG DNHS controls (22.6 L/min [15.6] vs 1.8 L/min [10.0]) (Fig. 1b). Six ambulatory participants treated with viltolarsen had PCF values > 160 L/min at Week 49.

**Fig. 2:**
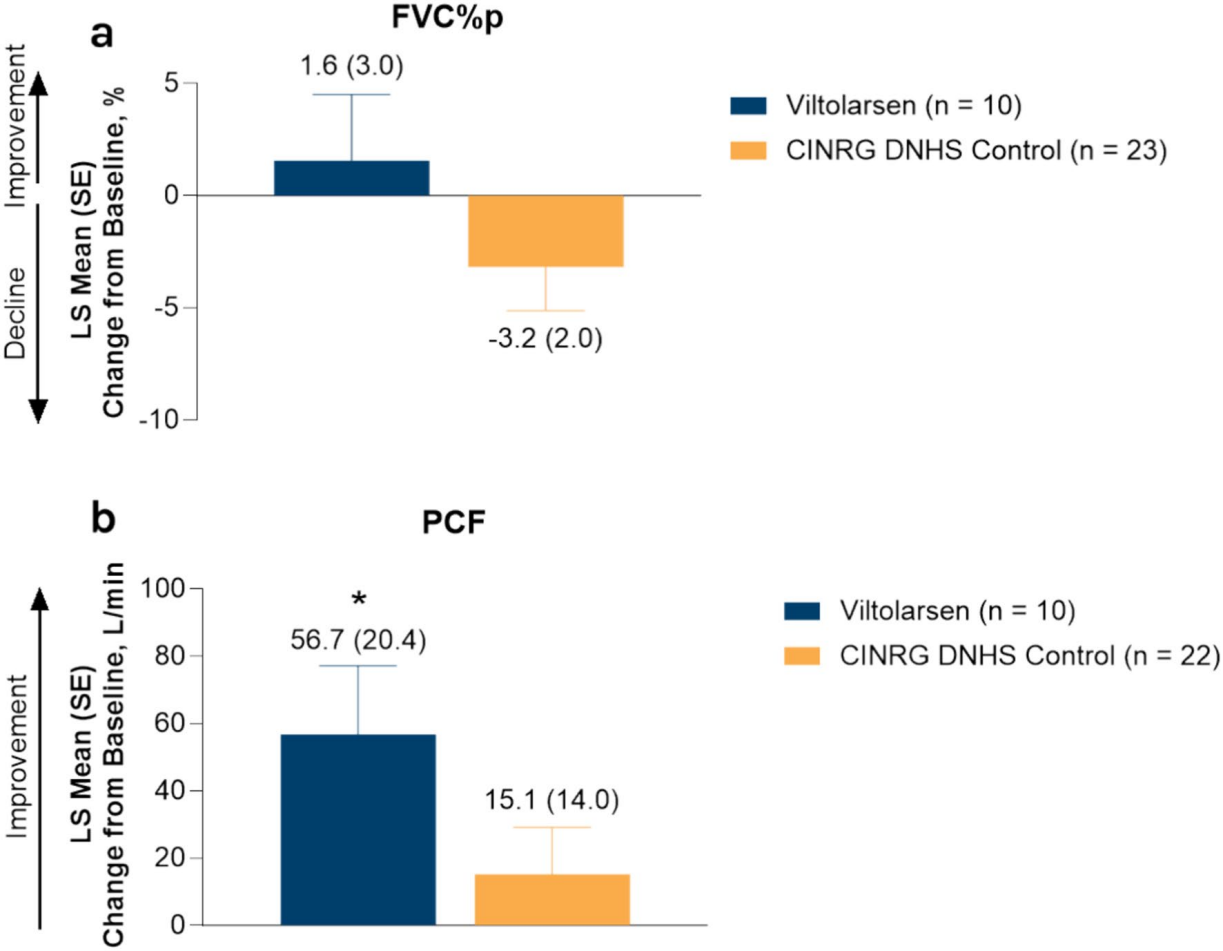
LS mean change from baseline in FVC%p (**a**) and PCF (**b**) in nonambulatory participants at Week 49. The values above the bars are LS mean (SE). All *P*-values are nominal. (**b**) *P = 0.01 vs baseline. *CINRG* Cooperative International Neuromuscular Research Group, *DNHS* Duchenne Natural History Study, *FVC%p* percent predicted forced vital capacity, *LS* least squares, *PCF* peak cough flow, *SE* standard error.

For nonambulatory participants, the LS mean change from baseline (SE) in FVC%p increased for participants treated with viltolarsen, whereas this value decreased for the CINRG DNHS control cohort (1.6% [3.0] vs − 3.2% [2.0]) (Fig. 2a). Furthermore, 90% (9/10) of nonambulatory participants receiving viltolarsen had an increase or stabilization in FVC%p from baseline, and 60% (6/10) of participants maintained FVC%p values > 50% at Week 49. The LS mean change from baseline (SE) in PCF for nonambulatory participants receiving viltolarsen vs the control cohort was 56.7 L/min (20.4) vs 15.1 L/min (14.0), respectively (Fig. 2b). Seven nonambulatory participants treated with viltolarsen had PCF values > 160 L/min at Week 49, including 3 participants with PCF values < 160 L/min at baseline that increased to > 160 L/min at Week 49.

**Fig. 3:**
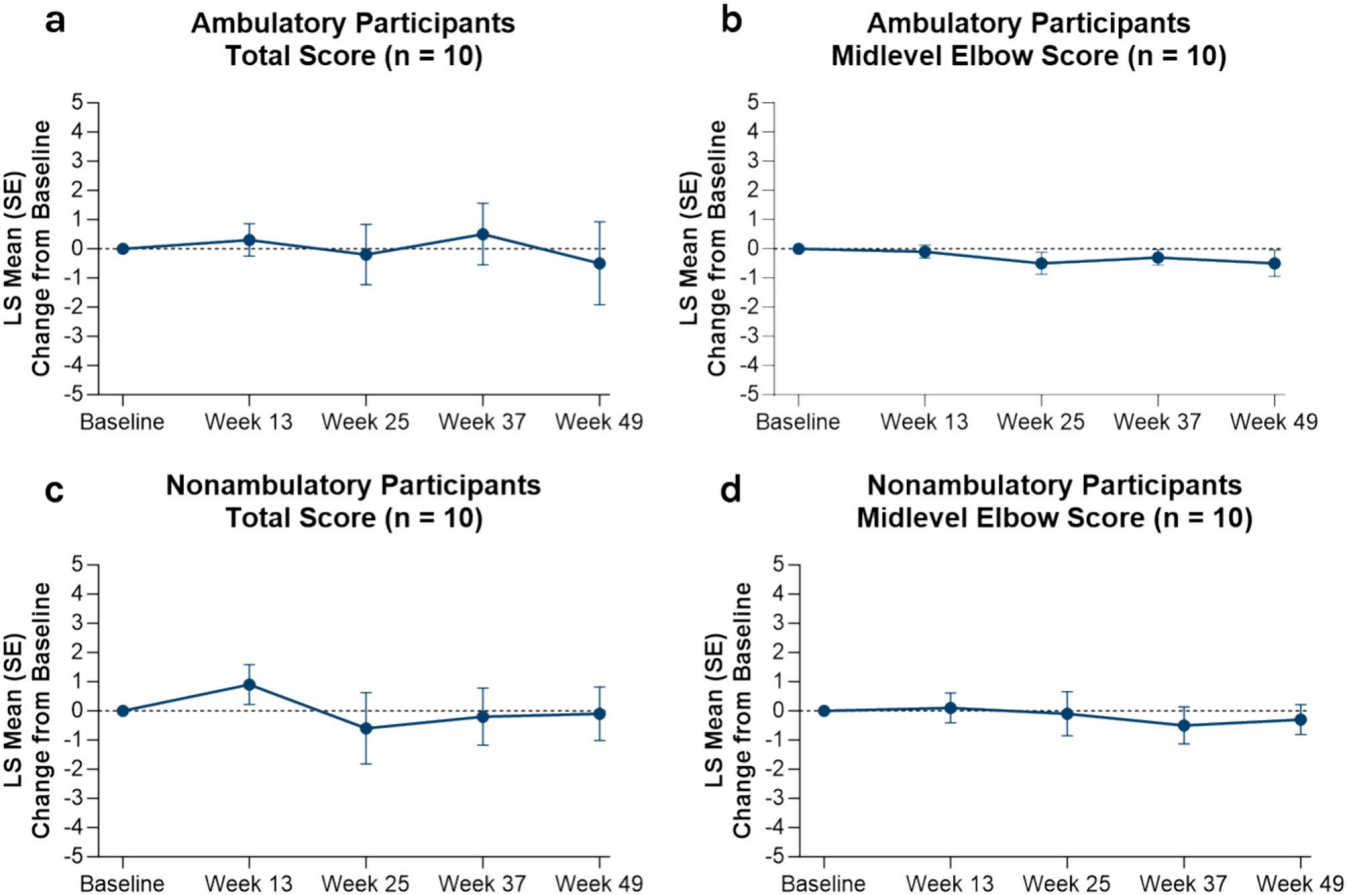
LS mean change from baseline in PUL 2.0 scores over time in ambulatory (**a,b**) and nonambulatory (**c,d**) participants receiving viltolarsen. Data not collected for CINRG DNHS control cohort. *CINRG* Cooperative International Neuromuscular Research Group, *DNHS* Duchenne Natural History Study, *LS* least squares, *PUL* Performance of Upper Limb, *SE* standard error.

Viltolarsen stabilized motor function in both ambulatory and nonambulatory males with DMD. The total and midlevel elbow scores from PUL 2.0 remained stable over 49 weeks regardless of ambulatory status (Fig. 3). The LS mean change (SE) from baseline in total North Star Ambulatory Assessment (NSAA) score at Week 49 for ambulatory participants was 2.2 points (0.8) for the viltolarsen group vs the CINRG DNHS cohort, whose value was − 2.5 points (1.4) (Fig. S1). The NSAA test was added later to the CINRG DNHS study protocol; therefore, only 3 participants had data to compare with the viltolarsen group.

## Discussion

There are limited approved treatment options for DMD that increase dystrophin protein production and provide symptomatic benefit, leaving an unmet need for patients with DMD. In the Galactic53 trial, the first trial of viltolarsen to evaluate pulmonary function, viltolarsen was well tolerated, showed clinical benefit in pulmonary function vs a control cohort, and stabilized motor function in both ambulatory and nonambulatory boys and older males with DMD amenable to exon 53 skipping.

TEAEs were reported by 95% (19/20) of participants receiving viltolarsen. All TEAEs were mild or moderate. The most frequent (occurring in ≥ 15% of participants) were COVID-19 infection, headache, hematuria, nasopharyngitis, and upper respiratory tract infection. There were no serious adverse events, deaths, or discontinuations due to TEAEs. Four participants experienced treatment-related TEAEs, and all were assessed as mild or moderate. No induction of serum anti-dystrophin or anti-viltolarsen antibodies was detected in participants receiving viltolarsen. Viltolarsen was well tolerated, and the safety profile in Galactic53 is consistent with previous phase I, phase II, and long-term extension studies of viltolarsen in boys with DMD^19–21^.

As the disease progresses, patients with DMD experience a loss of respiratory muscle strength that leads to complications and death^6,22^. Consequently, the American Thoracic Society (ATS) and the DMD Care Considerations Working Group guidelines both recommend that children with DMD have at least 1 baseline visit for pulmonary function testing between ages 4 and 6 years and twice-yearly visits after loss of ambulation and wheelchair dependence, a decrease in FVC%p below 80%, and/or age 12^6,22^. The recommended assessments at these visits include FVC and PCF, which were analyzed in this study, among others such as oxyhemoglobin saturation, maximal midexpiratory flow rate, and maximum inspiratory and expiratory pressures^22^.

A pulmonary assessment included in Galactic53, FVC%p, is particularly important, as its decline correlates with an increased risk for hospitalization^23^. Once values drop below 80% predicted, the risk of hospitalization increases, and this risk sequentially rises once the measure falls below 50%, 40%, and 30% predicted. Consequently, recommendations indicate to begin cough assist and nocturnal noninvasive ventilation when FVC%p is < 50% and daytime ventilation when FVC%p is < 20%^6,24^. In this study, viltolarsen provided meaningful pulmonary function benefits to both ambulatory and nonambulatory participants when compared with baseline and/or the CINRG DNHS control cohort. Least squares mean change from baseline in FVC%p was significantly higher for the ambulatory population receiving viltolarsen (mean age 9.8 years). For both ambulatory and nonambulatory populations, the majority (90%) of participants had FVC%p values that increased or were stable compared with baseline. These findings are clinically meaningful given that a 10% incremental decline in FVC%p is associated with a higher risk for hypoventilation^25^. In this study, all ambulatory participants and 6 of 10 nonambulatory participants remained above the 50% FVC%p threshold for needing cough assist and nocturnal ventilation interventions.

Effective airway clearance is a critical pulmonary function for patients with DMD, as it helps prevent pneumonia, hospitalization, respiratory failure, and death^22^. Peak cough flow values of < 160 L/min are associated with ineffective airway clearance^22^. Recommendations indicate that cough assist should be implemented when PCF values fall below 160 L/min and also when PCF values are below 270 L/min during acute respiratory illness^6,24^. In this study, participants treated with viltolarsen experienced larger increases in PCF compared with the CINRG DNHS control cohort. In addition, the PCF measurements of some nonambulatory participants that were below the recommended threshold for cough assist (< 160 L/min) at baseline improved to be above this threshold at Week 49. These data suggest that viltolarsen provides symptomatic benefits by improving or maintaining airway clearance in boys with DMD.

Taken together, the FVC%p and PCF values highlight the importance of viltolarsen as an early intervention to provide meaningful pulmonary function benefit and delay disease progression. Improvement in patient quality of life can be expected by delaying disease progression to the threshold at which assisted ventilation and cough function is needed. These factors may be particularly important for nonambulatory patients with DMD.

Without any pharmacological treatment, males with DMD lose ambulation and become wheelchair dependent at approximately age 8 to 12, and they continue to lose muscle strength over time^9,13,26^. As such, a key outcome of treatments for DMD should be their effect on motor function. In this study, viltolarsen stabilized upper limb motor function over 49 weeks for both ambulatory and nonambulatory participants as assessed by PUL 2.0 total and midlevel elbow scores. The midlevel elbow score reflects the ability to perform important tasks for patient quality of life and independence, such as moving weighted objects on a table, removing lids from containers, and lifting items to the mouth^27^. The stabilization of midlevel elbow scores by viltolarsen could have practical implications for males with DMD, such as maintaining the ability to feed themselves, brush their teeth, or open containers on their own. Ambulatory participants receiving viltolarsen also demonstrated improvement in NSAA total score over time while the control cohort score declined, and NSAA scores typically decline after age 7 for patients with DMD^28^. These results suggest that viltolarsen provides symptomatic benefit to motor function.

This study had limitations inherent to early phase studies and in studies of rare diseases, such as a small number of participants and the lack of a placebo control cohort. The use of the CINRG DNHS control cohort is appropriate for a phase II study of a rare disease, though it is less rigorous than a placebo-controlled trial. In Galactic53, the control cohort was matched to participants receiving viltolarsen on age and ambulatory and steroid status. Another limitation was that the selection by ambulatory status of the CINRG DNHS controls was not prespecified. Additionally, the CINRG DNHS control data was collected between 2006 and 2016, 5 years before Galactic53 began; however, there were no major changes in care guidelines for DMD during that period^6,26,29^. Furthermore, the NSAA was added later to the CINRG DNHS study protocol, limiting the data available for evaluation of the external control cohort. Also, PUL 2.0 was not assessed as part of the CINRG DNHS protocol, and therefore, there is no external control cohort to evaluate against the PUL 2.0 data collected in the Galactic53 clinical trial.

In conclusion, viltolarsen was well tolerated in both ambulatory and nonambulatory participants with DMD. Galactic53 is the first study to evaluate pulmonary function in males with DMD treated with viltolarsen and showed that both ambulatory and nonambulatory males treated with viltolarsen received clinical benefit in pulmonary function. Additionally, motor function was stabilized in ambulatory and nonambulatory participants. These results illustrate the age range and ambulation status over which viltolarsen can be considered an important part of the treatment strategy for patients with DMD amenable to exon 53 skipping.

## Methods

### Trial design and participants

Galactic53 was an open-label, phase II, multicenter study conducted at 8 sites in the US, China, Italy, Russia, Spain, and Turkey (NCT04956289; registered 09/07/2021). The study consisted of a Screening Period (Day − 28 to − 8), a Pre-infusion Period (Day − 7 to − 1), a Treatment Period where participants received once-weekly intravenous viltolarsen 80 mg/kg for 48 weeks, and a Follow-Up Period lasting 30 days (Fig. S2).

Eligible participants were males aged ≥ 8 years with a confirmed diagnosis of DMD with mutation(s) in the dystrophin gene amenable to exon 53 skipping. Participants were receiving a stable dose of glucocorticoids or were not treated with glucocorticoids for ≥ 3 months prior to the first dose of study drug and were expected to remain on a stable dose or off glucocorticoids for the duration of the study. A Brooke scale rating ≥ 3 or an upright FVC ≥ 30% were required for enrollment. The Brooke scale for upper limb function has grades ranging from 1 to 6. Grade 1 indicates the greatest range of motion and Grade 6 is assigned when a patient has no useful function of the hands.

Participants were excluded for evidence of symptomatic cardiomyopathy or if ventilation support was required while awake during the day. Participants with severe behavioral or cognitive problems that precluded participation, as judged by the investigator, were excluded. Participants were also excluded if they had asthma that required chronic treatment with a long-acting beta agonist. Other exclusion criteria included enrollment in a previous interventional study of viltolarsen and taking any other investigational drug or exon skipping agent within 3 months prior to the first dose of study drug or within 5 times the half-life of the medication, whichever was longer.

### Trial oversight

The trial protocol was approved by an institutional and/or licensing committee at each study site: Clinical Trial Ethics Committee of Hunan Children’s Hospital, Changsha City, China; The Third Medical Center of PLA General Hospital, Beijing, China; Comitato Etico della Fondazione Policlinico Universitario Agostino Gemelli IRCCS, Rome, Italy; Ethics Council of the Ministry of Health of the Russian Federation, Moscow, Russia; CEIC Fundacio Sant Joan de Deu Edifici Docent Sant Joan de Deu, Barcelona, Spain; Yeditepe Universitesi Klinik Arastirmalar Etik, Atasehir, Turkey; and Advarra, Columbia, USA. Prior to any study-related procedures, the participant provided written or verbal informed assent appropriate for age and development status and the participants’ parent or legal guardian provided written informed consent and/or HIPAA authorization. The trial was performed according to the principles of the Declaration of Helsinki and the Good Clinical Practice regulations. Trial activities were overseen by an independent data and safety monitoring board.

### Endpoints and assessments

The primary objective of the study was to determine the safety and tolerability of viltolarsen by recording TEAEs and serious adverse events. Secondary objectives included assessing pulmonary function, measured by FVC, and motor function, measured by PUL 2.0 and NSAA. An exploratory objective was to evaluate the strength of cough in participants with DMD by measuring PCF.

Assessment of FVC and PCF were measured on Day 1 (baseline), Week 13, Week 25, Week 37, and Week 49 (end of treatment). Baseline values for PUL 2.0 and NSAA were collected at the Pre-infusion Visit, and otherwise the schedule of these assessments was the same as the pulmonary assessments. FVC measures the maximum amount of air exhaled after maximum inhalation, and an expected value for FVC%p ranges from 80 to 120%^30^. FVC%p was considered stable if there was no change in values or an incremental decline of ≤ 10%. Cough strength measured by PCF for participants treated with viltolarsen was compared with CINRG DNHS controls at Week 49. The PUL 2.0 assessment measures the function of the upper limbs and includes a total score and 3 domain subscores (high level shoulder, midlevel elbow, and distal wrist and hand)^31^. Here we report PUL 2.0 total and midlevel elbow scores. The NSAA measures the motor function of ambulatory boys with DMD and consists of 17 items, such as standing, running, and head raising^32^. We report in the Supplement the total NSAA score in ambulatory participants receiving viltolarsen and the CINRG DNHS control cohort.

A master physiotherapist was responsible for providing good clinical practice (GCP)-compliant documentation of the inter-rater reliability and for training, retraining, and certification of each site clinical evaluator. The site clinical evaluator assessed study participants in accordance with GCP in performing the functional assessments as required per the protocol and documented the performance and results of such assessments.

### Statistics

The target sample size (N = 20) was based on the primary outcome of safety with a ≥ 95% probability to detect at least 1 adverse drug reaction with an incidence of 15%. Results from participants treated with viltolarsen were compared to controls from the CINRG DNHS^10^. Participants from the CINRG DNHS database were selected using the following criteria: had available baseline and Week 49 assessments for FVC, were within the enrollment age of the participants in Galactic53, had consistent steroid use defined as either not on steroids at baseline and Week 49 or on steroids at baseline and Week 49 with lifetime steroid use length up to the visit of at least 90 days, and met genetic eligibility criteria^19^. After initial patient selection was made, propensity score analysis was conducted before making the final data selection. This procedure included confounding variables of steroid status and ambulatory status and parameters of FVC, age, height, and weight at baseline.

PUL 2.0 was not assessed as part of the protocol for the CINRG DNHS study. The NSAA test was added later to the CINRG DNHS study protocol; therefore, only 3 participants had data to compare with the viltolarsen group.

For post hoc analyses, ambulatory and nonambulatory participants were matched separately. *P*-values derived from statistical analyses of these data are considered nominal.

The changes in FVC%p over the length of the study were compared between participants receiving viltolarsen and the historical control group using mixed models with repeated measures (MMRM). These models allowed the use of all available assessments per participant, included a random term per individual, and assessed the additional predictive value of a random slope term. As covariates of age and baseline value were included, the MMRM accounted for any imbalances between cohorts at baseline. The MMRM uses change from baseline (or % change from baseline) as the dependent variable; treatment, week of visit, and the treatment-by-week interaction as factors; and age and baseline value as covariates. The unstructured covariance model is used.

Descriptive statistics for PCF were generated at each time point that the assessment was performed. The change over time in scores was estimated in participants receiving viltolarsen using a MMRM. PCF values acted as the dependent variable, time was the independent variable, and a random term for individual was included.

## Supplementary Information

Supplementary Figures.

## Data Availability

The data generated/analyzed during the current study are available upon reasonable request from the corresponding author or the study sponsor (trialinfo@nspharma.com).
