# Supplementary material for: Safety and efficacy of viltolarsen in ambulatory and nonambulatory males with Duchenne muscular dystrophy
Source: Sci Rep. 2024 Oct 8;14:23488. doi: 10.1038/s41598-024-70783-y (PMC11461856; doi:10.1038/s41598-024-70783-y)
Supplement: Supplementary file 1 — Supplementary Figures. [file 41598_2024_70783_MOESM1_ESM.pdf]

Supplementary Information

Safety and efficacy of viltolarsen in ambulatory and nonambulatory males with Duchenne muscular dystrophy

Amy D. Harper[1], Haluk Topaloglu[2], Eugenio Mercuri[3], Vasiliy Suslov[4], Liwen Wu[5], Cigdem Y. Ayanoglu[2], Michael Tansey[6], Michelle L. Previtera[6], Robert A. Crozier[6], Leslie Magnus[6], Paula R. Clemens[7,8]

[1]Children's Hospital of Richmond at Virginia Commonwealth University, Richmond, VA, USA; [2]Department of Pediatrics, Yeditepe University, Istanbul, Turkey; [3]Gemelli Hospital Catholic University Foundation, Roma, Italy; [4]Saint Petersburg State Paediatric Medical University, St Petersburg, Russia; [5]Hunan Children's Hospital, Hunan, China; [6]NS Pharma, Inc., Paramus, NJ, USA; [7]Department of Neurology, University of Pittsburgh School of Medicine, Pittsburgh, PA, USA; [8]Department of Veterans Affairs Medical Center, Pittsburgh, PA, USA

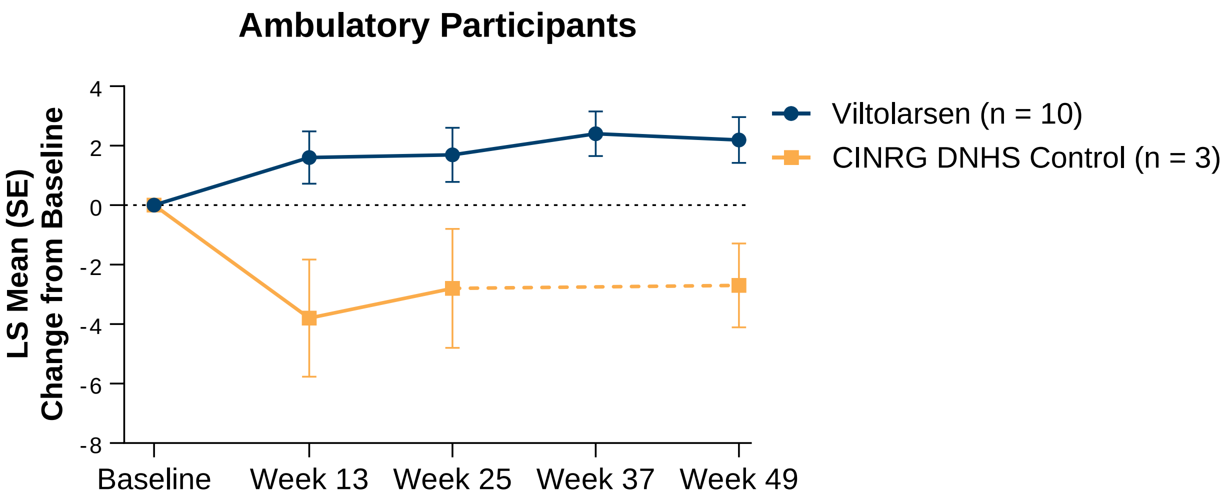

Figure S1. LS mean change from baseline in NSAA total score over time.

NSAA total score (mean [SD]) at baseline was 16.8 (7.9) for the viltolarsen group (n = 10) and 20.0 (12.5) for the CINRG DNHS group (n = 3).

CINRG, Cooperative International Neuromuscular Research Group; DNHS, Duchenne Natural History Study; LS, least squares; NSAA, North Star Ambulatory Assessment; SD, standard deviation; SE, standard error.

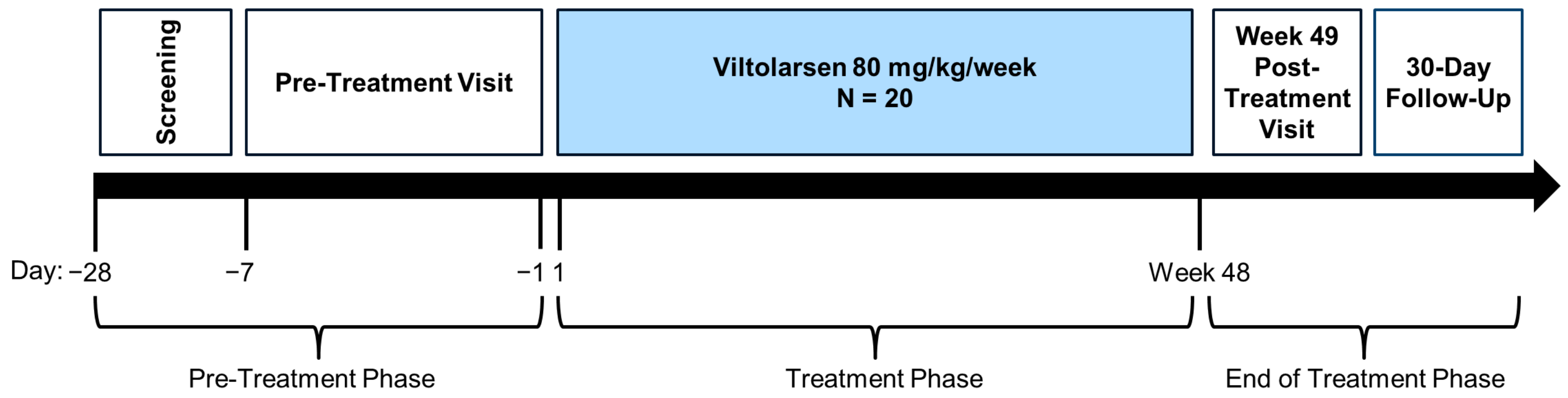

Figure S2. Galactic53 study design.
